# Tissue-Protective Mechanisms of Bioactive Phytochemicals in Flap Surgery

**DOI:** 10.3389/fphar.2022.864351

**Published:** 2022-04-25

**Authors:** Andrea Weinzierl, Emmanuel Ampofo, Michael D. Menger, Matthias W. Laschke

**Affiliations:** Institute for Clinical and Experimental Surgery, Saarland University, Homburg, Germany

**Keywords:** phytochemicals, nutraceuticals, herbal medicine, flap, necrosis, ischemia–reperfusion injury

## Abstract

Despite careful preoperative planning, surgical flaps are prone to ischemic tissue damage and ischemia–reperfusion injury. The resulting wound breakdown and flap necrosis increase both treatment costs and patient morbidity. Hence, there is a need for strategies to promote flap survival and prevent ischemia-induced tissue damage. Phytochemicals, defined as non-essential, bioactive, and plant-derived molecules, are attractive candidates for perioperative treatment as they have little to no side effects and are well tolerated by most patients. Furthermore, they have been shown to exert beneficial combinations of pro-angiogenic, anti-inflammatory, anti-oxidant, and anti-apoptotic effects. This review provides an overview of bioactive phytochemicals that have been used to increase flap survival in preclinical animal models and discusses the underlying molecular and cellular mechanisms.

## Introduction

The preparation of tissue flaps is one of the central surgical techniques in plastic surgery, with numerous options and variations in use, covering a broad spectrum of indications. The primary goal of flap surgery is usually to reconstruct missing tissue and to restore the form and function of the body, but secondary benefits can contribute to the decision to perform flap surgery. For instance, well-perfused muscle tissue can be a crucial factor in combatting tissue infections, such as osteomyelitis, in addition to the obliteration of the dead space and the reconstruction of the soft tissue coverage (Zhang et al., 2021).

Flap surgery is, however, associated with several complications, such as wound dehiscence, wound breakdown, or partial necrosis, with an incidence of up to ∼50% (Fischer et al., 2013). These ischemia-induced complications are favored by the fragile microcirculation of tissue flaps. In fact, during flap elevation, a large portion of the perfusing vessels is severed, for e.g., when an adipocutaneous flap is dissected from the underlying richly perfused fascia. Accordingly, the tissue has to adapt to the changed blood supply and the decreased arterial inflow. Flap areas distal to this inflow are left ischemic when capillary perfusion pressure is no longer maintained above the critical level, which triggers cellular hypoxic signaling pathways, leading to apoptosis or necrosis (Kalogeris et al., 2014; Lucas, 2017). In the case of random pattern flaps, blood perfusion is provided by the dermal plexus and/or musculocutaneous arterioles passing through the flaps’ base. Accordingly, the flaps are planned, considering certain length-to-width ratios depending on their tissue composition and preparation site (Saint-Cyr et al., 2009; Memarzadeh et al., 2016). Additional factors that contribute to the changes in tissue perfusion after flap elevation are manifold and include the effects of sympathetic denervation, local inflammatory changes, elevated interstitial pressure, and the neovascularization of the flap from the wound bed (Lucas, 2017). Therefore, mechanisms other than ischemic cell death can contribute to flap tissue damage. For instance, the changed flow rates and pro-inflammatory mediators cause a prothrombotic environment that can lead to capillary obstruction, both by microthrombosis and by vessel occlusion from the invading immune cells (Menger et al., 2003).

During free flap transfer, the tissue additionally has to endure a period of complete ischemia, while the flap is moved from the donor to the recipient site and vascular anastomosis is performed. After the reestablishment of blood perfusion, tissue reoxygenation can paradoxically result in additional cell damage, also referred to as ischemia–reperfusion injury (IRI), due to reactive oxygen species (ROS) formation and leukocytic inflammation (Siemionow and Arslan, 2004). To overcome ischemic cell damage and flap tissue IRI, several approaches have been developed in the past decades. In addition to invasive strategies, such as surgical delay Reinisch (1974) and ischemic preconditioning Zahir et al., (1998), pharmacological treatment with phytochemicals is an emerging concept to improve tissue survival.

**FIGURE 1:**
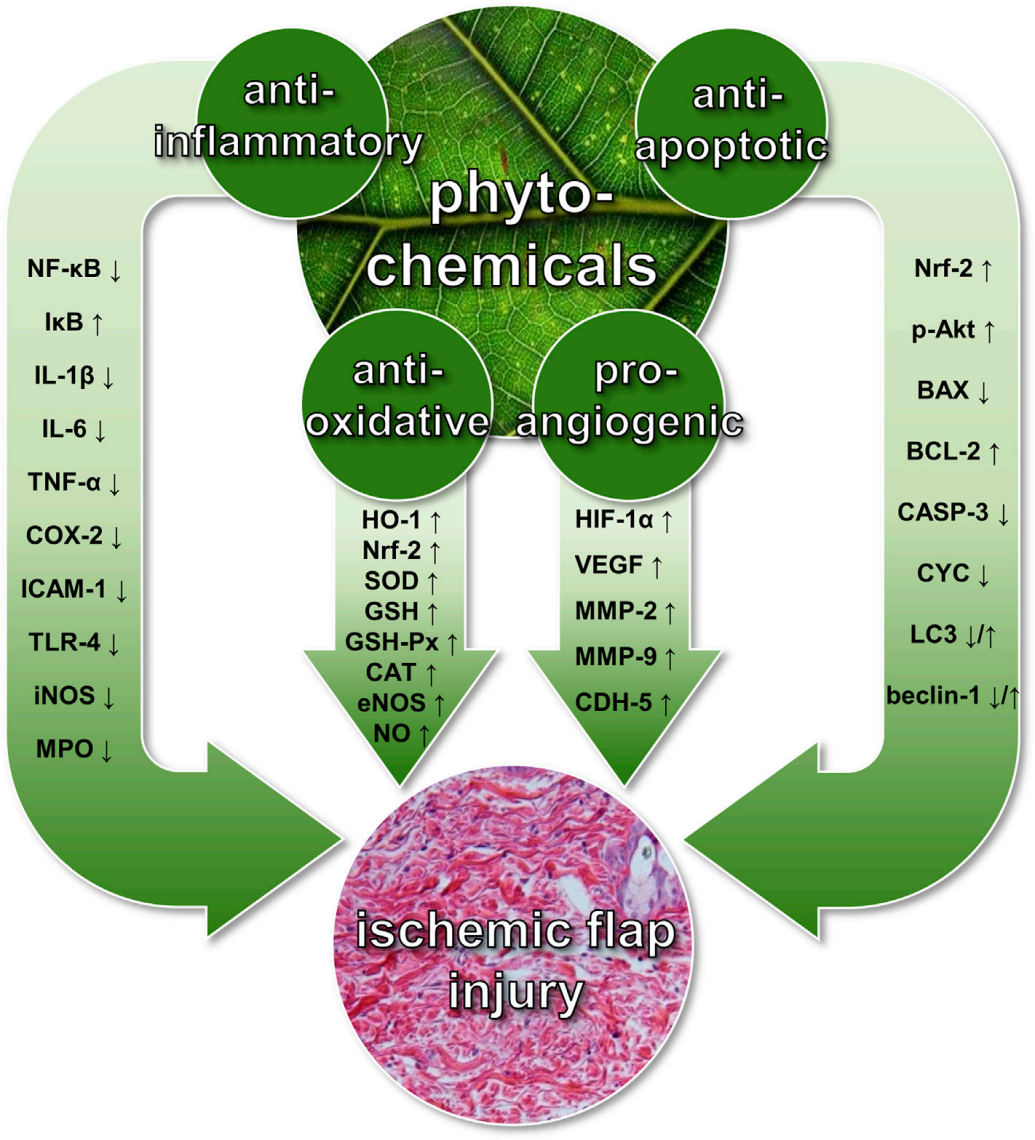
Beneficial effects of phytochemicals on ischemic flap injury. These include pro-angiogenic, anti-inflammatory, anti-oxidant, and anti-apoptotic mechanisms, which are mediated by the up- or downregulation of various signaling molecules. P-Akt = phosphorylated protein kinase B; BAX = B-cell lymphoma 2-associated X protein; BCL-2 = B-cell lymphoma 2; CASP-3 = caspase-3; CAT = catalase; CDH-5 = cadherin-5; COX-2 = cyclooxygenase-2; CYC = cytochrome C; eNOS = endothelial nitric oxidase synthase; GSH = glutathione; GSH-Px = glutathione peroxidase; HIF-1α = hypoxia-inducible factor-1α; HO-1 = hemeoxygenase-1; ICAM-1 = intercellular adhesion molecule-1; IL-1β/IL-6 = interleukin-1β/interleukin-6; iNOS = inducible nitric oxide synthase; IκB = nuclear factor of kappa light polypeptide gene enhancer in B-cells inhibitor; LC3 = microtubule-associated proteins 1A/1B light chain 3B; MMP-2/MMP-9 = matrix metalloproteinase-2/matrix metalloproteinase-9; MPO = myeloperoxidase; NF-κB = nuclear factor kappa-light-chain-enhancer of activated B cells; NO = nitric oxide; Nrf-2 = nuclear factor erythroid 2-related factor-2; SOD = superoxide dismutase; TLR-4 = toll-like receptor-4; TNF-α = tumor necrosis factor-α; VEGF = vascular endothelial growth factor.

Phytochemicals are defined as bioactive plant-derived compounds that are not considered essential to the human diet (Liu, 2013). Compounds derived from mushrooms or mycelia are usually also included in this general category, even though they are not plants. Although rapidly increasing experimental evidence suggests the efficacy of phytochemical compounds for the prevention and therapy of various pathologies (George et al., 2021; Ismaeel et al., 2021), their potential is still underestimated in clinical practice. Particularly in flap surgery, phytochemicals may be highly promising to prevent ischemic tissue damage because they promote the formation of new microvessels (Thangapazham et al., 2016) and are capable of modulating anti-inflammatory, anti-oxidant, and anti-apoptotic signaling pathways (Figure 1) (Haidarali et al., 2015; Zhang et al., 2015; Al-Ishaq et al., 2020). Furthermore, they have been shown to modulate the tissue microenvironment (Cheng et al., 2016), i.e., the tissue’s exact molecular and cellular composition, including growth factors and cytokines, all cell types, and the extracellular matrix. Of interest, this tissue microenvironment is also tightly associated with flap survival (Vogt et al., 2005; Pei et al., 2020). In line with this view, we provide in the present review for the first time an up-to-date overview of experimental studies analyzing the beneficial effects of phytochemicals on blood perfusion and tissue survival of surgical flaps.

## Introductory Remarks

In the context of phytochemical flap treatment, we have mainly identified alkaloid, terpenoid, and phenolic compounds. Alkaloids are a group of cyclic nitrogen-containing compounds that are found in over 20% of plant species (Kennedy and Wightman, 2011). Many poisons, neurotoxins, and traditional psychedelics (e.g., atropine and scopolamine), and also most social drugs (e.g., nicotine, caffeine, and cocaine) consumed by humans are part of this chemical group (Zenk and Juenger, 2007). Terpenoids are a group of lipid-soluble compounds that are present in *Ginkgo biloba* and ginseng extracts. They have been shown to act synergistically, which may explain the fact that it is difficult to identify a major single active component in many terpenoid-containing herbal extracts, complicating their standardization (Kennedy and Wightman, 2011). Last, phenolics are ubiquitously found across the plant kingdom. Flavonoids, a subgroup of phenolics, are particularly well represented in this review because they are well-known anti-oxidant and anti-inflammatory mediators (Panche et al., 2016).

Most phytochemicals do not only target one specific mechanism within the cell but rather modulate several pathways at once with synergistic effects adding up to the overall outcome. This pleiotropic mode of action makes it difficult to trace all altered mechanisms and to identify the primary cause for the observed outcome. In line with this fact, almost all addressed studies describe various effects of the examined phytochemical compounds without specifying one dominant mechanism. These effects are summarized for isolated compounds or whole plant extracts in Table 1. However, to improve the readability of the present review and to avoid unnecessary repetitions, individual phytochemicals may only be discussed in the context of one selected mode of action. Furthermore, compounds, such as sea buckthorn extract or acai seed extract, have been included in this review for the sake of comprehensiveness, even though the corresponding studies include little or even no analysis to clarify the underlying mechanisms of flap necrosis reduction (Emsen, 2005; Coelho da Mota et al., 2018).

**TABLE 1:** Overview of isolated phytochemical compounds and whole plant extracts that have been evaluated in experimental flap models. Availability and selected mechanisms mediating their effects are listed.

Compound	Selected mechanism				Flap model	Reference
	*Angiogenesis*	*Apoptosis*	*Inflammation*	*Oxidative stress*		
Apigenine	VEGF↑	—	IL-1β↓, IL-6↓, and TNF-α↓	SOD↑	Caudally based, dorsal random pattern flap in rats	Ma et al., (2021)
Asiaticoside	VEGF↑	—	IL-1β↓, IL-6↓, and TNF-α↓	SOD↑	Caudally based, dorsal random pattern flap in rats	Feng et al., (2021)
Astragaloside IV	VEGF↑	LC3↑ and beclin-1↑	IL-1β↓, IL-6↓, and TNF-α↓	SOD↑	Caudally based, dorsal random pattern flap in rats	(Lin et al., 2017)
Azadirachtin A	VEGF↑	—	NF-kB↓, IL-1β↓, IL-6↓, TNF-α↓, and TLR-4↓	—	Caudally based, dorsal random pattern flap in rats	He et al., (2020)
Baicalein	VEGF↑	CASP-3↓	—	SOD↑ and GSH↑	Caudally based, dorsal random pattern flap in rats	Lin et al. (2018b)
Betulinic acid	VEGF↑, MMP-9↑, and CDH-5↑	BAX↓, CYC↓, CASP-3↓, LC3II↑, and beclin-1↑	—	HO-1↑, SOD↑, and eNOS↑	Caudally based, dorsal random pattern flap in mice	Li et al., (2019)
Butylphthalide	VEGF↑ and CDH-5↑	BAX↓, CYC↓, CASP-3↓, beclin-1↓, and LC3↓	—	HO-1↑, SOD↑, and eNOS↑	Dorsal multi-territory perforator flap in rats	Li et al., (2021)
Caffeic acid phenethyl ester	—	—	—	NO↑	Inferior epigastric artery flap in rats; 11 h ischemia	Bilen et al., (2006)
	—	—	—	SOD↑, GSH↑, and GSH-Px↑	Caudally based, dorsal random pattern flap in rats	Aydogan et al., (2007)
Crocin	VEGF↑	—	—	SOD↑	Caudally based, dorsal random pattern flap in rats	Cai et al., (2020)
Curculigoside A	VEGF↑	—	—	SOD↑	Caudally based, dorsal random pattern flap in rats	Chen et al., (2018b)
Epigallocatechin gallate	VEGF↑	—	—	—	Caudally based, dorsal random pattern flap in rats	Cheon et al., (2012)
	—	—	TNF-α↓	SOD↑ and GSH-Px↑	Inferior epigastric artery flap in rats; 0, 3, 6, 9, and 12 h ischemia	Aslan et al., (2014)
Genistein	VEGF↑	BCL-2↑		SOD↑	Cranially based, dorsal random pattern flap in rats	Fáber et al., (2018)
Ginkgolide B	VEGF↑	BAX↓, BCL-2↑, and CASP-3↓	—	HO-1↑, Nrf-2↑, and SOD↑	Dorsal multi-territory perforator flap in rats	Lin et al., (2019b)
Hydroxysafflor yellow A	VEGF↑ and MMP-9↑	BAX↓, BCL-2↑, CASP-3↓, LC3II↓, and beclin-1↓	—	HO-1↑, SOD↑, GSH↑, and eNOS↑	Dorsal multi-territory perforator flap in rats	Zhang et al., (2020a)
	CDH-5↑					
Icariin	VEGF↑	—	IL-1β↓, Il-6↓, and TNF-α↓	SOD↑	Caudally based, dorsal random pattern flap in rats	Huang et al., (2019)
Kaurenoic acid	—	—	IL-1β↓, TNF-α↓, and MPO↓	GSH↑	Caudally based, dorsal random pattern flap in rats	de Lima Silva et al., (2015)
Leonurine	VEGF↑	CASP-3↓	—	SOD↑	Dorsal multi-territory perforator flap in rats	Lin et al., (2020)
Luteolin	—	p-Akt↑, BAX↓, BCL-2↑, and CASP-3↓	IL-1β↓, IL-6↓, TNF-α↓, and MPO↓	HO-1↑ and SOD↑	Inferior epigastric artery flap in rats; 4 h ischemia	Chen et al., (2018b)
Naringin	VEGF↑	—	IL-6↓ and TNF-α↓	SOD↑	Caudally based, dorsal random pattern flap in rats	Cheng et al., (2017a)
Nobiletin	VEGF↑	—	—	SOD↑	Caudally based, dorsal random pattern flap in rats	Jiang et al., (2020)
Resveratrol	VEGF↑, MMP-9↑, and CDH-5↑	BAX↓, CYC↓, CASP-3↓, LC3II↑, and beclin-1↑	—	HO-1↑, SOD↑, GSH↑, and eNOS↑	Caudally based, dorsal random pattern flap in rats	Lin et al., (2019b)
Salidroside	VEGF↑	BAX↓, BCL-2↑, and CASP-3↓	IL-6↓ and TNF-α↓	SOD↑	Caudally based, dorsal random pattern flap in rats	Deheng et al., (2016)
Salvianolic acid B	VEGF↑ and MMP-2↑	—	—	—	Inferior epigastric artery flap in rats; 3 h ischemia	Lay et al., (2003)
	VEGF↑, MMP-9↑, and CDH-5↑	BAX↓, CYC↓, CASP-3↓, LC3II↑, and beclin-1↑	—	HO-1↑, SOD↑, GSH↑, and eNOS↑	Caudally based, dorsal random pattern flap in rats	Lin et al., (2018a)
Tetramethylpyrazine	VEGF↑	p-Akt↑, Nrf-2↑, BAX↓, and BCL-2↑	—	—	Dorsal multi-territory perforator flap in rats	Qing et al., (2019)
Thymoquinone	—	—	—	Mitochondrial damage↓	Caudally based, dorsal random pattern flap in rats	Kocak et al., (2016)

Plant extract	Selected mechanism				Flap model	Reference
	*Angiogenesis*	*Apoptosis*	*Inflammation*	*Oxidative stress*		
Acai seed extract	Perfused vessel density↑	—	—	—	Transverse rectus abdominis muscle flap in hamsters	Coelho da Mota et al., (2018)
*Antrodia camphorata* extract	VEGF↑	—	NF-κB↓, IκB↑, IL-6↓, TNF-α↓, iNOS↓, and COX-2↓	—	Cranially based, dorsal random pattern flap in mice	Tsai et al. (2015)
Black soybean seed coat extract	—	—	NF-κB↓, p-IκBα↓	—	Inferior epigastric artery flap in rats; 10 h ischemia	Kim et al., (2012)
			COX-2↓, and ICAM-1↓			
Copaiba oil	—	—	MPO↓	GSH↑	Caudally based, dorsal random pattern flap in rats	De Lima Silva et al., (2009)
Grape seed extract	Vessel formation score↑	—	—	Neutrophil infiltration score↓	Inferior epigastric artery flap in rats; 8 h ischemia	Karaaslan et al., (2010)
Olive oil	VEGF↑	—	—	—	Caudally based, dorsal random pattern flap in rats	Kirkil et al., (2016)
Radix astragali extract	VEGF↑	—	—	SOD↑	Caudally based, dorsal random pattern flap in rats	Cai et al., (2015)
Red ginseng extract	VEGF↑	—	—	—	Caudally based, dorsal random pattern flap in rats	Yun et al., (2017)
	HIF-1α↑	—	—	CAT↑	Caudally based, dorsal random pattern flap in rats	Tekin et al., (2017)
Rosemary extract	Vessel diameter↑	—	—	—	Cranially based, dorsal random pattern flap in rats	İnce et al., (2016)
	VEGF↑	—	IL-1↑ and TNF-α↓	—	Cranially based, dorsal random pattern flap in rats	İnce et al., (2018)
Sanguis draconis	VEGF↑	—	—	SOD↑	Abdominal perforator flap in rats	Zhang et al., (2016)
Sea buckthorn extract	—	—	—	—	Caudally based, dorsal random pattern flap in rats	Emsen, (2005)

Akt/p-Akt protein kinase B, phosphorylated protein kinase B; BAX, B-cell lymphoma 2-associated X protein; BCL-2, B-cell lymphoma 2; CASP-3, caspase 3; CAT, catalase; CDH-5, cadherin 5; COX-2, cyclooxygenase 2; CYC, cytochrome C; eNOS, endothelial nitric oxidase synthase; GSH, glutathione; GSH-Px, glutathione peroxidase; HIF-1α, hypoxia-inducible factor 1α; HO-1, hemeoxygenase 1; ICAM-1, intercellular adhesion molecule 1; IL-1β/IL-6, interleukin 1β/interleukin 6; iNOS, inducible nitric oxide synthase; IκB/p-IκBα, nuclear factor of kappa light polypeptide gene enhancer in B-cells inhibitor/phosphorylated IκB α; LC3, microtubule-associated proteins 1A/1B light chain 3B; MMP-2/MMP-9, matrix metalloproteinase 2/matrix metalloproteinase 9; MPO, myeloperoxidase; NF-κB, nuclear factor kappa-light-chain-enhancer of activated B cells; NO, nitric oxide; Nrf-2, nuclear factor erythroid 2-related factor 2; SOD, superoxide dismutase; TLR-4, toll-like receptor 4; TNF-α, tumor necrosis factor α; VEGF, vascular endothelial growth factor.

**FIGURE 2:**
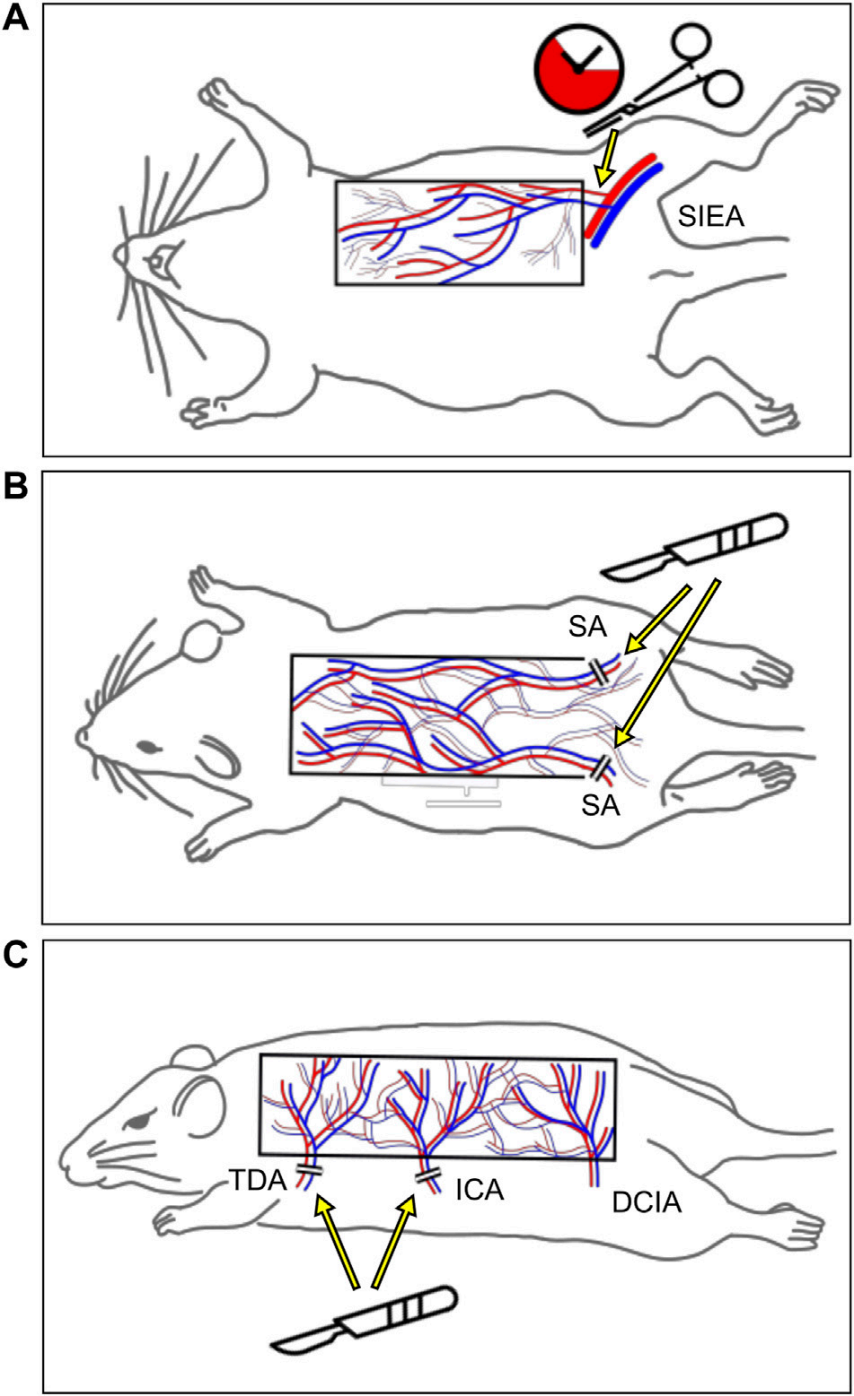
Schematic of the three most frequently used animal flap models. **(A)** The superficial inferior epigastric artery flap enables the transient interruption of the blood flow into the flap with a clap. **(B)** The random pattern “McFarlane” flap is designed on the dorsum of the animal. In the majority of studies, the flap base is located caudally and both sacral arteries (SAs) are ligated to ensure distal flap necrosis. **(C)** The more recently established multi-territory dorsal perforator flap includes the supply area of the thoracodorsal (TDA), intercostal (ICA), and deep circumflex iliac artery (DCIA). By raising the flap on the vascular axis of the DCIA, this model simulates the physiology of perforator flaps.

Similarly, as few experimental flap models (Figure 2) have been redundantly used to analyze the effects of phytochemicals; these models are not discussed for every individual study included in this review but are listed in Table 1. Of note, studies focusing on IRI and its prevention are typically conducted in models with a period of total ischemia and subsequent reperfusion, such as the axial epigastric island flap (Figure 2A) (Petry and Wortham, 1984; Padubidri and Browne, 1997). Moreover, many studies are performed on well-established random pattern flap models, which are simple to prepare and are characterized by high reproducibility (Figure 2B) (McFarlane et al., 1965; Schmauss et al., 2018). A third popular flap preparation is based on the deep circumflex iliac artery as the axial vessel and contains several perforasomes to simulate the physiology of perforator flaps (Figure 2C) (Saint-Cyr et al., 2009; Wang et al., 2017). However, even if the same experimental flap model is applied in individual studies, differences in lab animals, surgical techniques, flap dimensions, or duration of ischemia are likely to influence flap survival rates. Hence, direct comparisons between individual studies should be considered with caution.

## Effects of Phytochemicals on Surgical Flaps

We have identified four general mechanisms underlying the beneficial effects of phytochemicals on blood perfusion and tissue survival of surgical flaps. These include pro-angiogenic, anti-inflammatory, anti-oxidant, and anti-apoptotic mechanisms, as outlined in the following sections.

### Pro-Angiogenic Mechanisms

Angiogenesis, i.e., the development of new microvessels from pre-existing ones, is of major importance for flap survival. This process is regulated by a balanced interplay of pro- and anti-angiogenic factors and requires the close interaction of endothelial cells and perivascular cells (Chiaverina et al., 2019). Blood vessel development is typically driven by tissue exposure to hypoxia or inflammatory cytokines in the peripheral flap tissue (Schürmann et al., 2014). Under ischemic conditions, hypoxia-inducible factor (HIF)-1α is no longer degraded by proteasomes and translocates into the nucleus, in which it induces the expression of vascular endothelial growth factor (VEGF), the central cytokine stimulating angiogenesis (Zimna and Kurpisz, 2015). Almost all studies presented in this review (Table 1) reported an increased VEGF expression in flap tissue, including studies focusing on crocin, curculigoside A, epigallocatechin gallate, nobiletin, olive oil, rosemary extract, or sanguis draconis (Cheon et al., 2012; Kirkil et al., 2016; Zhang et al., 2016; Chen T. et al., 2018; İnce et al., 2018; Cai et al., 2020; Jiang et al., 2020). However, only Korean red ginseng extract has also been proven to upregulate HIF-1α as the underlying mechanism (Yun et al., 2017).

*In vitro*, it has been shown that the phytochemical radix astragali extract stimulates endothelial cell proliferation and motility via the VEGF axis (Zhang et al., 2009). In line with these findings, Cai et al. (2015) could increase the survival of random pattern flaps in radix astragali extract-treated rats. In a follow-up study using the identical flap model, Lin et al. (2017) suggested that this effect may be mediated by astragaloside IV as one of the pro-angiogenic molecules in this root extract.

Additional examples of phytochemicals with a high pro-angiogenic activity promoting flap survival are resveratrol (Ciloglu et al., 2014; Lin J. et al., 2019), betulinic acid (Li et al., 2019), salvianolic acid B (Lay et al., 2003; Lin J. et al., 2018), and hydroxysafflor yellow A (Zhang C. et al., 2020). All of these compounds simultaneously increase the expression of VEGF and matrix metalloproteinases (MMPs). During the process of angiogenesis, the latter enzyme group is responsible for the degradation of the basement membrane surrounding each blood vessel, allowing new vessel sprouts to form (Quintero-Fabián et al., 2019).

### Anti-Inflammatory Mechanisms

In response to tissue injury, cytokines are crucial in initiating and sustaining an inflammatory reaction with the goal of reestablishing tissue integrity (Zhang et al., 2006). However, in the case of ischemic flaps, this inflammatory reaction rather exacerbates the stress on the hypoxic tissue by releasing ROS and increasing endothelial permeability, resulting in the formation of tissue edema (van den Heuvel et al., 2009). The release of inflammatory cytokines and the invasion of immune cells, particularly neutrophilic granulocytes, is therefore a hallmark of flap-associated IRI (Hernandez et al., 1987).

Several studies reported a downregulation of the expression of the pro-inflammatory transcription factor nuclear factor (NF)-κB, for instance, after the application of *Antrodia camphorata* extract or black soybean seed coat extract (Kim et al., 2012; Tsai et al., 2015). Other works analyzed the expression and secretion of inflammatory cytokines in flap tissue after the administration of phytochemicals. As expected, the analyzed phytochemicals exerted a suppressive effect on these cytokines, which was associated with an improved flap survival (Table 1). For instance, tumor necrosis factor (TNF)-α and interleukin (IL)-6 serum levels were significantly lower in random pattern flaps and axial epigastric island flaps of salidroside-, luteolin-, and naringin-pretreated rats when compared to controls (Deheng et al., 2016; Cheng L. et al., 2017; Chen G. et al., 2018). Moreover, apigenine, asiaticoside, icariin, and astragaloside IV inhibited inflammation in ischemic random pattern flaps, as demonstrated by a reduced expression of TNF-α, IL-1β, and IL-6 (Lin et al., 2017; Huang et al., 2019; Feng et al., 2021; Ma et al., 2021). Another compound that suppresses tissue inflammation following flap elevation is azadirachtin A. In fact, He et al. (2020) demonstrated a markedly reduced secretion of TNF-α and IL-1β in random pattern flap tissue of azadirachtin A-treated animals. Of interest, the authors additionally found a downregulation of toll-like receptor (TLR)-4, which is a central receptor known to trigger tissue injury during IRI by aggravating the inflammatory response (Lee et al., 2016).

### Anti-Oxidant Mechanisms

ROS formation by ischemic cells or invading neutrophilic granulocytes play a detrimental role in damaging flap tissue (Suzuki et al., 1991). While a certain amount of ROS is necessary for adequate cellular signaling and the regulation of apoptosis, the excessive presence of ROS and other free radicals can damage the tissue (Sies, 2015). Once the anti-oxidant defenses of the cells are exhausted, ROS start to disrupt physiological cellular mechanisms by causing DNA strand breaks or by oxidizing, thus inactivating proteins (Neri et al., 2015).

It is well known that phytochemicals exert anti-oxidant effects by functioning as free radical scavengers or by modulating signaling pathways responsible for ROS homeostasis and anti-oxidant gene expression (Zhang et al., 2015; Cheng YT. et al., 2017). The latter mechanism is mainly mediated by the transcription factor nuclear factor erythroid 2-related factor (Nrf)-2, which regulates the expression of anti-oxidant response elements, such as hemeoxygenase (HO)-1 and superoxide dismutase (SOD)-1 (Sotolongo et al., 2020). Several studies reported that resveratrol increases the expression of HO-1 in an Nrf-2-dependent manner (Gao et al., 2018; Zhang Y. et al., 2020; Lian et al., 2020). In line with this finding, the administration of resveratrol elevated the expression of HO-1 and SOD-1 in random pattern flaps of rats, which was associated with a lower apoptosis rate (Ciloglu et al., 2014; Lin J. et al., 2019). Moreover, luteolin and ginkgolide B triggered the gene expression of Nrf-2, leading to elevated HO-1 mRNA levels and higher viability of surgical flaps (Chen G. et al., 2018; Lin D. et al., 2019). A significant upregulation of HO-1 in flaps has also been observed after the administration of betulinic acid, salvianolic acid B, and hydroxysafflor yellow A (Lin J. et al., 2018; Li et al., 2019; Zhang C. et al., 2020). HO-1 also modulates the expression of the enzyme endothelial nitric oxide synthase (eNOS) (Luo et al., 2018). This induction of eNOS counteracts ROS, which consume its product, the vasodilator NO. In addition to its anti-oxidant effects, NO also inhibits the endothelial adhesion of leukocytes and their subsequent tissue invasion (Kubes et al., 1991; Ma et al., 1993). These beneficial effects can be observed after the application of betulinic acid, butylphthalide, hxydroxysafflor yellow A, resveratrol, and salvianolic acid B to flap tissue (Lin J. et al., 2018, Lin et al., 2019 J.; Li et al., 2019, 2021; Zhang C. et al., 2020).

Another anti-oxidant enzyme that is upregulated after phytochemical application is glutathione peroxidase (GSH-Px) (Table 1). Its upregulation or the upregulation of the substrate glutathione (GSH) can be observed after the administration of many phytochemicals discussed in this review, including caffeic acid phenethyl ester, kaurenoic acid, and copaiba oil (Aydogan et al., 2007; De Lima Silva et al., 2009; de Lima Silva et al., 2015).

### Anti-Apoptotic Mechanisms

Several phytochemicals exert their tissue-protective effects on ischemic flap tissue by modulating signaling pathways of apoptosis. One of them is tetramethylpyrazine (TMP), an alkaloid compound that has long been used in traditional Chinese medicine for the treatment of cardiovascular and cerebrovascular diseases (Donkor et al., 2016). Qing et al., (2019) reported that TMP attenuates apoptosis in the ischemic areas of dorsal multi-territory perforator flaps in rats due to a decreased ratio of the pro-apoptotic protein B-cell lymphoma protein (BCL)-2-associated X protein (BAX) and the anti-apoptotic protein BCL-2. In addition, the authors detected reduced protein kinase B (Akt) phosphorylation, indicating that TMP may suppress cell death by interfering with this major pro-apoptotic pathway (Qing et al., 2019).

Chen G. et al., (2018) found that the administration of luteolin, a naturally occurring polyphenol found in several vegetables, ameliorates IRI-induced flap tissue injury by inhibition of acute inflammation and lowering oxidative stress levels. The investigation of the underlying signaling revealed that luteolin preconditioning decreases the cytotoxic effect of H_2_O_2_ in keratinocytes (Chen G. et al., 2018). This is due to increased phosphorylation of Akt, an upregulated expression of BCL-2, and a downregulated expression of BAX (Chen G. et al., 2018). Similarly, the phenolic compound salidroside improved the survival of random pattern skin flaps in rats (Deheng et al., 2016). This was associated with an enhanced BCL-2 expression and diminished expression of BAX and cleaved caspase (CASP)-3 in the flap tissue (Deheng et al., 2016). Moreover, flaps in baicalein- and leonurine-treated animals exhibited decreased levels of CASP-3 (Lin R. et al., 2018; Lin et al., 2020).

Another mechanism, by which phytochemicals counteract apoptosis, is the modulation of autophagy. As a highly conserved survival mechanism of all eukaryotic cells, autophagy primarily acts as an adaptive response to cellular stressors, such as nutrient deprivation and/or ischemia (Webster, 2012). During autophagy, the cell starts to degrade and recycle macromolecules, including proteins, lipids, and carbohydrates, for the synthesis of essential components and as an energy supply (Glick et al., 2010). The beneficial effect of autophagy on flap survival could be observed in a study by Lin J. et al., (2018), who investigated the effects of salvianolic acid B on random pattern skin flap necrosis. They found an upregulated expression of microtubule-associated protein 1A/1B-light chain 3 (LC3)-II as well as beclin-1 and vacuolar sorting protein (VSP) 34 in areas of ischemia, indicating higher numbers of autophagosomes and autolysosomes (Lin J. et al., 2018). More importantly, they detected a suppression of classical apoptosis markers, such as CASP-3, cytochrome C (CYC), and BAX. This may be explained by the fact that autophagy and apoptosis act complementary to each other. Autophagy blocks the induction of apoptosis, while caspase activation downregulates the autophagic process (Mariño et al., 2014). Similarly, studies evaluating the phytochemicals such as betulinic acid, resveratrol, and astragaloside IV for the prevention of flap necrosis found that upregulating autophagy plays a central role in increasing tissue survival (Lin et al., 2017, Lin et al., 2019 J.; Li et al., 2019). These compounds enhanced the expression of LC3-II and beclin-1 and decreased ischemic cell death within surgical flaps. These findings demonstrate the beneficial effect of phytochemical-induced autophagy on flap tissue survival. In contrast, the treatment of rat multi-territory perforator flaps with hydroxysafflor yellow A and butylphthalide increased flap tissue survival and downregulated the autophagy markers LC3 and beclin-1 (Zhang C. et al., 2020; Li et al., 2021). Nonetheless, both compounds also suppressed apoptotic cell death, as shown by a reduced number of CASP-3-positive cells in the ischemic tissue. Hence, the authors speculated that excessive autophagy may lead to self-digestion and eventually cell death (Zhang C. et al., 2020). Taken together, these contradictory results indicate that additional studies are required to further unravel the effects of phytochemical-induced autophagy on ischemic flap injury.

## Discussion

The medicinal use of phytochemicals has a long history dating back more than 5,000 years. Phytochemicals are still present as health resources today because particularly in developing countries they are more readily available than prescription drugs (Aggarwal et al., 2007). Accordingly, around 80% of people worldwide are still relying on herbal medicines for some part of primary healthcare (Ekor, 2014). Moreover, more and more phytochemicals are also used in Western medicine as useful and effective therapeutic substances (Siddiqui et al., 2015).

Many phytochemical compounds have been shown to effectively improve flap survival in preclinical animal studies, as outlined in the present review. However, the exact mechanisms underlying this beneficial effect usually remain elusive. This is most probably due to the fact that phytochemicals typically exhibit a pleiotropic spectrum of activity, simultaneously targeting different molecular and cellular processes. Although this may contribute to their efficiency, it makes it difficult to develop approved clinical treatment regimens because it hampers the assessment of the clinical response to their application and associated side effects by clear readout parameters. Hence, extensive preclinical studies focusing on the identification of the phytochemicals’ main mode of action in highly standardized *in vitro* and *in vivo* settings are largely required. Moreover, robust data on the pharmacokinetics and pharmacodynamics of individual phytochemicals need to be assessed. In addition, it is important to analyze possible interactions between phytochemicals and conventional drugs, as their combined use may bear the risk of unwanted complications (Nowack, 2008; Li et al., 2017). On the other hand, the supplementation of pharmacotherapy with phytochemicals offers the opportunity to improve the efficiency of current treatment modalities while reducing the dosage of individual components and thus their side effects. In this context, it may also be beneficial to evaluate different routes of phytochemical administration. For instance, the topical application of phytochemicals on flap tissue may help prevent systemic side effects. For this purpose, future studies should clarify which phytochemicals fulfill the biopharmaceutical requirements for effective topical drug delivery, particularly through the skin barrier (Gugleva et al., 2021).

Clinical studies focusing on phytochemicals in flap surgery are still rare, even though the favorable risk–benefit profile of phytochemicals would make them good candidates for the treatment of patients (Wahab et al., 2021). Indeed, from the compounds addressed, only three have been tested in human studies in the context of flap surgery, i.e., salvianolic acid B, TMP, and hydroxysafflor yellow A (Chen et al., 2010; Ma et al., 2010; Shining and Zha, 2011). Interestingly, the studies on hydroxysafflor yellow and TMP focused on the positive effect of the compounds on blood rheology, while the blood flow characteristics were not analyzed in the respective preclinical studies (Ma et al., 2010; Shining and Zha, 2011). Similarly, Chen et al., (2010) speculated that the effect of *Salvia miltiorrhiza* extract and its component salvianolic acid B is largely based on the inhibition of platelet aggregation and increase in prothrombin time. This observation highlights the fact that it is difficult to identify and monitor all involved pathways due to the pleiotropic mode of action of phytochemicals.

Furthermore, many phytochemicals remain largely unstandardized. In fact, source plants can markedly differ in their phytochemical content (Chen et al., 2019). In addition, the variable composition of available products, including teas or extracts, makes it difficult to obtain consistent data on their toxicological risk profile (Collins et al., 2020). Hence, whenever possible, the use of phytochemicals with a well-defined content, such as *Ginkgo biloba* extract EGb 761 (Bekerecioǧlu et al., 1998; Christen and Maixent, 2002; Sambuy et al., 2012) or purified plant compounds, may contribute to the generation of robust data and thus the establishment of evidence-based phytochemical treatment strategies. In fact, such standardized or purified compounds may be crucial to prove the efficiency and safety in comparison to conventional therapeutic approaches in randomized controlled multicenter trials. In this context, it should also be considered that many phytochemical compounds may not be patentable or their patents have expired a long time ago. Hence, there is no incentive for the pharmaceutical industry to drive the development in this field or even to conduct expensive clinical trials. Therefore, the synthesis of chemical analogs of phytochemicals may be an alternative route to consider. This may also increase their therapeutic potential, as it would overcome the limited availability of phytochemicals in their natural sources (Mora-Pale et al., 2013).

The generally missing translation of preclinical studies into human trials may be caused by several critical factors, such as the poor solubility and stability of phytochemicals and their lack of selectivity. For instance, low solubility may cause poor absorption into targeted cells. Moreover, the activity of phytochemical compounds gets compromised due to the methods of their extraction and purification. Phytochemicals usually also exhibit a comparatively low bioavailability that complicates their application (Holst and Williamson, 2008). Modifications in the extraction/purification protocols for phytochemicals or the use of natural bioavailability enhancers may help to overcome these problems (Singh et al., 2019; Asuzu et al., 2022). In addition, there are several promising strategies for targeted drug delivery, such as the generation of micronized and encapsulated formulations or the binding of phytochemicals to nanocarriers (Smoliga and Blanchard, 2014). In fact, there has been a continually increasing interest in generating nanoformulations of phytochemicals by using liposomes, micelles, nanoemulsions, and nanoparticles to maximize their therapeutic potential (Singh et al., 2019). However, unresolved issues related to nanotechnology, such as the toxicity of nanomaterials, their degradability, stability, and selectivity, also need to be carefully evaluated before using them for the application of phytochemicals (Sharifi et al., 2012; Allen, 2019).

Another important factor, which may crucially determine the successful integration of phytochemical treatment into perioperative routines, is the time point of treatment application. For instance, Fáber et al. (2018) reported that the administration of genistein starting 3 days prior to flap elevation is more effective than an administration starting the day of flap elevation. Similarly, the administration of thymoquinone before and after flap elevation decreases flap necrosis more effectively than isolated pre- or postoperative treatment (Kocak et al., 2016). These findings clearly demonstrate that the identification of the most effective treatment sequence is of major importance for the clinical application of phytochemicals in flap surgery.

Taken together, a rapidly increasing number of preclinical studies suggest that phytochemicals bear great potential for the improvement of surgical flap survival due to their pleiotropic, tissue-protective effects. The next important step toward the clinical application of such phytochemicals in flap surgery is the evaluation of their efficiency and safety in prospective, randomized clinical trials. If this succeeds, the application of phytochemicals may markedly prevent ischemic flap complications and improve the overall outcome of flap-based reconstructive procedures in future clinical practice.
